# Type 2 Diabetes Mellitus with Diabetic Ketoacidosis, Hypertriglyceridemia, and Acute Pancreatitis in an Adolescent with Autism Spectrum Disorder and Pancreatic Divisum

**DOI:** 10.7759/cureus.76818

**Published:** 2025-01-02

**Authors:** Tsubasa Adachi, Shinji Higuchi, Tomohisa Okuma, Jun Mori

**Affiliations:** 1 Division of Pediatric Endocrinology, Metabolism and Nephrology, Children's Medical Center, Osaka City General Hospital, Osaka, JPN; 2 Department of Diagnostic Radiology, Osaka City General Hospital, Osaka, JPN

**Keywords:** acute pancreatitis, autism spectrum disorder, diabetic ketoacidosis, hypertriglyceridemia, pancreatic divisum, type 2 diabetes mellitus

## Abstract

Children with developmental disorders such as autism spectrum disorder (ASD) may not only develop type 2 diabetes mellitus (T2DM) due to psychosocial stress and overeating but also experience severe complications such as acute pancreatitis (AP) and hypertriglyceridemia (HTG). Consequently, in pediatric patients with concurrent T2DM and developmental disorders, a comprehensive approach is necessary that includes not only imaging evaluations for AP but also assessments of risk factors such as psychological stress and metabolic abnormalities. We report the case of a 13-year-old male child, with a family history of T2DM in his paternal grandfather, who presented with severe diabetic ketoacidosis (DKA) and HTG (triglycerides 2118 mg/dL). His condition was considered to have been triggered by psychosocial stress following the divorce of his parents two months previously, which led to episodes of overeating. Two weeks prior to admission, he had consumed excessive amounts of soft drinks. The patient was initially treated with fluids, insulin, and mannitol for cerebral edema. On the third day post admission, he developed AP, which was confirmed by the occurrence of abdominal pain, elevated pancreatic enzyme levels, and the findings of CT imaging. Subsequent imaging revealed pancreatic divisum. The patient was also diagnosed with ASD during hospitalization. Following a temporary initial recovery, the patient experienced worsening obesity and was started on metformin and icosapent ethyl to manage recurrent T2DM and HTG. In this case, the development of T2DM was considered to have been primarily associated with ASD, which subsequently led to DKA, HTG, and AP, with pancreatic divisum believed to be an additional predisposing factor contributing to these conditions. To the best of our knowledge, there have been no previous reports of T2DM associated with DKA, HTG, AP, ASD, and pancreatic divisum.

## Introduction

Diabetic ketoacidosis (DKA) is a severe acute complication of diabetes mellitus, characterized by hyperglycemia, metabolic acidosis, and ketosis/ketonuria. In adults, DKA may also be associated with the further complication of acute pancreatitis (AP), with an incidence of 10-15% [1,2], although its incidence in children has yet to be determined. AP is, nevertheless, a potentially lethal disease with varying degrees of severity and an overall mortality of 4.8% in adults, whereas the incidence of severe AP can be as high as 13.5% [3]. Early detection and appropriate treatment are accordingly essential.

In children, the causes of AP include trauma, anatomical abnormalities, medications, infections, gallstones, hyperlipidemia, idiopathic factors, and systemic illnesses, such as diabetic acidosis, genetic diseases, and autoimmune diseases [4]. To date, although there have been reports of the occurrence of type 2 diabetes mellitus (T2DM) with DKA, hypertriglyceridemia (HTG), and AP [5,6], there have, to the best of our knowledge, been no reported cases of T2DM accompanied by DKA, HTG, AP, autism spectrum disorder (ASD), and pancreatic divisum. Herein, we describe a case involving a child with ASD and pancreatic divisum who resorted to excessive consumption of food and sugary beverages following the divorce of his parents, leading to obesity and the subsequent development of T2DM, DKA, HTG, and AP.

This report was presented as an abstract at the 2023 Annual Meeting of the Japanese Pediatric Society on April 14, 2023.

## Case presentation

The patient was a 13-year-old boy with no exceptional medical history. His family history included T2DM in his paternal grandfather. Following the divorce of his parents, the boy had not attended school for two months, and during this time, he had consumed excessive amounts of solid food at home. In contrast, in the two weeks prior to a visit to a local hospital, he had rarely eaten solid food, although he had drunk large quantities of soft drinks (4-5 L/day). He was initially brought to the local hospital with chest and back pain and had gradually become somnolent.

The results of laboratory tests revealed metabolic acidosis (pH 7.098, bicarbonate 7.4 mmol/L, base excess -20.7), severe hyperglycemia (>600 mg/dL), and ketonuria (3+). Additional findings included amylase, lipase, plasma osmolality, and insulin levels of 74 IU/L, 35 U/L, 414 mOsm/kg H_2_O, and 1.94 μIU/mL, respectively. Based on these findings, the patient was diagnosed with DKA and initially treated with intravenous fluids and insulin infusion. However, he was subsequently transferred to our pediatric intensive care unit (PICU) on account of impaired consciousness.

On admission to the PICU, a physical examination revealed Kussmaul's respiration and acanthosis nigricans. He was 170 cm tall, weighed 86 kg (compared with a pre-illness weight of 94 kg), and had a body mass index of 29.9. The results of laboratory analyses indicated severe hyperglycemia (1228 mg/dL), metabolic acidosis (pH 7.062, bicarbonate 3.0 mmol/L, base excess -25.9), dehydration-related renal dysfunction (blood urea nitrogen (BUN) 39.5 mg/dL, creatinine 1.12 mg/dL, uric acid 14.6 mg/dL), hyperosmolarity (375 mOsm/kg H_2_O), and HTG (2118 mg/dL) (Table 1). The diabetes workup revealed glycated hemoglobin (HbA1c) of 16.8%, elevated 3β-hydroxybutyrate (12880 μmol/L), and low serum C-peptide (1.63 ng/mL at a blood glucose concentration of 701 mg/dL), with urine C-peptide at 95.7 μg/day. There was an absence of autoantibodies against diabetes mellitus, although a computed tomography (CT) scan of the head revealed cerebral edema. These findings accordingly confirmed the diagnosis of DKA secondary to T2DM, on the basis of which, he was treated with bowel rest, intravenous fluids, continuous insulin infusion to gradually lower the elevated blood glucose levels, head elevation for cerebral edema, and electrolyte correction.

**Table 1:** Laboratory data at admission PT-INR: prothrombin time-international normalized ratio; GAD: glutamic acid decarboxylase; IA-2: Islet antigen 2

Laboratory parameters	Patient values	Reference range
pH	7.062	7.350-7.450
Partial pressure of oxygen (PaO2)	131	83-108 mmHg
Partial pressure of carbon dioxide (PaCO2)	11	35-48 mmHg
Bicarbonate (HCO3⁻)	3.0	22-26 mmol/L
Base excess (BE)	-25.9	-2-+2 mmol/L
Lactate	11	5-14 mg/dL
WBC	23210	3580-8150 /μL
Neutrophil	75.3	39.6-67.0%
RBC	6.06	4.29-5.70 10^6/μL
Hemoglobin	16.2	13.3-16.6 g/dL
Platelet count	281	172-359 10^3/μL
Aspartate aminotransferase (AST)	13	8-38 U/L
Alanine transaminase (ALT)	14	4-44 U/L
Lactate dehydrogenase (LDH)	274	124-222 U/L
Gamma-glutamyl transpeptidase (γ-GTP)	45	16-73 U/L
Total protein	7.1	6.7-8.3 g/dL
Albumin	4.1	3.9-4.9 g/dL
Triglyceride	2118	50-149 mg/dL
High-density lipoprotein (HDL) cholesterol	35	40-90 mg/dL
Low-density lipoprotein (LDL) cholesterol	95	70-139 mg/dL
Amylase	78	41-112 U/L
Blood urea nitrogen (BUN)	39.5	8.0-20.0 mg/dL
Creatinine	1.12	0.60-1.10 mg/dL
Uric Acid	14.6	3.7-7.0 mg/dL
Sodium	139	138-145 mEq/L
Potassium	5.7	3.6-4.8 mEq/L
Chloride	100	101-108 mEq/L
Calcium	9.7	8.8-10.2 mg/dL
Phosphorus	5.0	1.8-2.4 mg/dL
C-reactive protein (CRP)	0.25	<0.26 mg/dL
Plasma osmolality	375	275-295 mOsm/kg H_2_O
Activated partial thromboplastin time (APTT)	20.6	9.9 sec
PT-INR	0.97	0.90-1.10
Blood glucose	1228	73-109 mg/dL
Glycated hemoglobin (HbA1c)	16.8	4.6-6.2%
3β-Hydroxybutyric acid	12880	μmol/L
Serum C-peptide	1.63	0.80-2.50 ng/mL
24-hour urinary C-peptide	95.7	μg/day
Anti-GAD antibody	negative	negative
Anti-IA-2 antibody	negative	negative
Anti-insulin antibody	negative	negative

**Figure 1:**
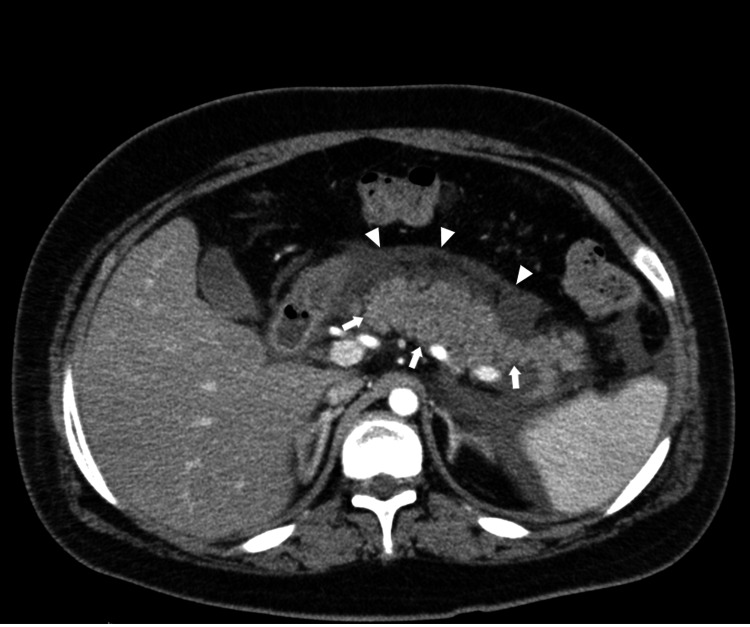
Arterial phase abdominal dynamic CT showing pancreatic enlargement (white arrow), an enhanced density of retroperitoneal adipose tissue, and retroperitoneal fluid retention (white arrow heads) CT showed no areas of poor contrast in the pancreatic parenchyma, dilatation of the main pancreatic duct, or calcified stones.

After two days of treatment, improvement was noted in his state of consciousness and levels of blood glucose and acidosis. However, on day 3, increases were detected in the levels of the inflammatory marker, C-reactive protein (CRP) (17.02 mg/dL) and amylase (625 U/L), and he developed abdominal pain. Contrast-enhanced abdominal CT confirmed pancreatic enlargement and the presence of peripancreatic fluid, consistent with AP, although no gallstones were detected (Figure 1). Further laboratory tests revealed elevated levels of pancreatic amylase (346 U/L), lipase (483 U/L), trypsin (8260 ng/mL), and elastase-1 (3904 ng/dL), consistent with AP (Table 2).

**Table 2:** Laboratory data at the onset of pancreatitis

Laboratory parameters	Patient values	Reference range
Amylase	625	41-112 U/L
Pancreatic Amylase	346	18-53 U/L
Lipase	483	17-57 U/L
Trypsin	8260	100-550 ng/mL
Elastase-1	3904	0-300 ng/dL

The patient was subsequently treated with bowel rest, intravenous fluid, and antimicrobial prophylaxis (meropenem, 1 g/day), and by day 7, the abdominal symptoms had resolved, pancreatic enzyme levels were normalized, and abdominal CT revealed a reduction in pancreatic swelling and no evidence of pancreatic cysts or infections. Consequently, enteral feeding was initiated, intravenous insulin was discontinued, and insulin aspart and glargine were administered. On day 8, the patient was transferred to a general ward, and during the subsequent period of recovery, his fat intake was gradually increased, insulin levels were adjusted, and exercise and nutritional therapy were initiated, with insulin aspart and glargine being discontinued on days 15 and 19, respectively.

The patient had not previously been diagnosed with ASD. However, during the hospitalization, he exhibited limited empathy and shared interactions with others, reduced eye contact, minimal facial expressions, and few friendships. Additionally, he demonstrated restricted interests and hypersensitivity to certain sounds. The patient was ultimately diagnosed with ASD by a psychiatrist, and to enhance their understanding of this condition, the patient and his family were provided with information regarding the characteristics of ASD. In addition, relevant environmental adjustments were made, including home visits by relatives and health nurses. Treatment approaches for ASD include behavioral therapy and pharmacological interventions. Behavioral therapy, however, is typically suitable for individuals of an age and motivation level that allows them to engage effectively, which was not applicable to the patient. Regarding pharmacological treatment, medications such as risperidone and aripiprazole are commonly used for children with ASD. However, these medications are known to have side effects, including increased appetite, which posed a significant concern for the patient. As a result, their use was deemed unsuitable and ultimately abandoned. The patient was discharged on day 51 of hospitalization. 

**Figure 2:**
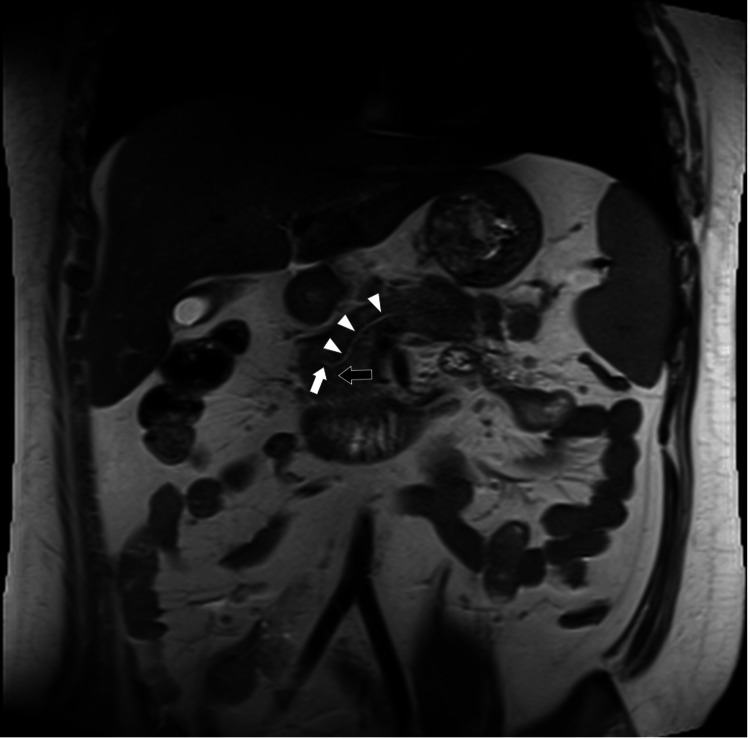
T2-weighted coronal image of incomplete pancreatic divisum showing dorsal pancreatic duct (white arrow) connecting to the ductus Santorini (white arrow heads) and opening into the accessory pancreatic duct. The ventral pancreatic duct (black arrow) is thin and considered an incomplete type of pancreatic divisum.

In this case, the findings of abdominal magnetic resonance imaging (MRI) and magnetic resonance cholangiopancreatography (MRCP) imaging provided evidence to indicate pancreatic divisum (Figure 2). Although the patient's blood glucose and triglyceride levels returned to normal in response to initial treatment, his obesity-associated symptom subsequently worsened, resulting in a recurrence of T2DM and HTG. Consequently, metformin and icosapent ethyl were initiated, and with the consent of the patient and his father, genetic testing for hyperchylomicronemia was performed, although no pathogenic variants were detected. At the time of writing, there has been no recurrence of AP.

## Discussion

In the reported case, the development of T2DM was considered to be primarily associated with ASD, with subsequent progression to DKA, HTG, and AP. The patient had pre-existing ASD and pancreatic divisum that were diagnosed during hospitalization. However, following the divorce of his parents, he resorted to overeating, thereby exacerbating his obese condition and contributing to the development of T2DM, DKA, HTG, and AP. AP develops as a consequence of both organic conditions and psychosocial factors, thereby highlighting the necessity for a comprehensive evaluation assessing organic diseases and psychosocial factors in managing cases of T2DM complicated by AP.

To the best of our knowledge, there have been no previous reports of T2DM associated with DKA, HTG, AP, ASD, and pancreatic divisum. However, given that it is conceivable that the cases of other patients with similar conditions have previously been overlooked, we do not necessarily intend to imply that cases such as this are rare. 

Although the focus in cases of AP associated with T2DM or HTG often centers on metabolic causes, the potential occurrence of structural abnormalities in the pancreas also warrants consideration. Accordingly, even though such abnormalities were not the primary pathology in the present case, we recommend a thorough evaluation of pancreatic anatomy using MRI and MRCP. Reports have indicated that between 1.5% and 25.9% of pediatric cases of AP may involve structural abnormalities of the pancreas, such as pancreatic divisum or abnormal union of the pancreaticobiliary junction [4]. Pancreatic divisum, which arises because of a failure of fusion between the ventral and dorsal pancreatic ducts, has been identified as the most common congenital anomaly of the pancreatic ductal system, which is observed in 4-10% of the general population and is implicated in 12-50% of cases of idiopathic AP in children [7]. However, it is essential to note that the presence of pancreatic divisum does not necessarily lead to AP [8]. In the present case, given that there had been no previous symptoms indicative of pancreatitis, pancreatic divisum is considered to have been a secondary contributing factor to the development of AP. We consulted a pediatric surgeon concerning this case. We determined that endoscopic retrograde cholangiopancreatography or surgical intervention should be considered if pancreatitis recurs without exacerbating metabolic factors. 

The details of this case highlight the importance of considering AP, particularly HTG, as a complication of DKA, given that HTG or chylomicronemia accounts for 1-7% of AP cases [9]. In this context, lipoprotein lipase (LPL), expressed in the capillary endothelial cells of muscles and adipose tissue, hydrolyzes triglycerides, yielding glycerol and fatty acids and plays a key role in regulating fat metabolism by catalyzing the breakdown of chylomicrons [10]. The pathophysiology of HTG is associated with elevated levels of free fatty acids, microcirculatory dysfunction, oxidative stress, and Ca2+ overload [9]. Treatments include fluid resuscitation, pain management, initial bowel rest, and specific interventions, such as insulin therapy, heparin, plasma exchange, and hemofiltration, to lower triglyceride levels [11]. Furthermore, it has been established that cases of HTG in which the serum levels of triglycerides exceed 500 mg/dL are associated with a heightened risk of AP [12], and elevated levels of triglycerides may also be associated with the severity and prognosis of AP [13]. In the present case, upon admission, the patient's triglyceride level was 2118 mg/dL, thereby indicating a high risk of subsequent AP development. Ultimately, however, owing to treatment with DKA, the patient fasted whilst receiving insulin and heparin, which contributed to a rapid alleviation of HTG.

Given that in patients with DKA, the initial symptoms of AP can be overlooked, physicians should pay close attention to this condition. Abdominal pain is reported by patients in 46% of DKA cases [14] and is commonly reported in cases of acute AP. DKA may also be associated with altered states of consciousness, thereby sometimes making it difficult for patients to report abdominal pain. Accordingly, when managing patients with DKA, physicians should confirm the occurrence of abdominal pain and regularly monitor the serum levels of amylase, lipase, and triglycerides. 

The patient described in this report, diagnosed with ASD, had experienced the divorce of his parents during his adolescence, which is assumed to have contributed to his refusal to attend school and overeating. It is believed that his excessive consumption of sugary beverages led to the onset of DKA, a condition referred to as "soft drink-related DKA." Although the association between ASD and DKA has yet to be conclusively determined, children with ASD have been established to consume excessive quantities of sugary soft drinks [15]. Moreover, compared with those without ASD, individuals with ASD are at a higher risk of developing T2DM [16]. Consequently, when managing ASD patients with T2DM, it is necessary to adopt a multidisciplinary approach that addresses not only organic diseases but also psychosocial issues.

## Conclusions

In the case reported, the development of T2DM was considered to be primarily associated with ASD, which subsequently led to the development of DKA, HTG, and AP. Pancreatic divisum is believed to be an additional predisposing factor contributing to these conditions. To the best of our knowledge, this is the first report of a case of T2DM associated with DKA, HTG, AP, ASD, and pancreatic divisum.

Children with developmental disorders such as ASD may not only develop T2DM due to psychosocial stress and overeating but also experience severe complications such as acute pancreatitis and hypertriglyceridemia. In pediatric patients with developmental disorders, it is essential to base assessments on a comprehensive approach that includes imaging-based assessments for conditions such as AP and evaluations of risk factors, including psychological stress and metabolic abnormalities. Adopting this type of holistic strategy will contribute to addressing the underlying and contributory factors, thereby ensuring a more effective management of the disease's metabolic and psychosocial aspects.
